# Distinctive development of embryo and endosperm caused by male gametes irradiated with carbon-ion beam

**DOI:** 10.1007/s00497-024-00496-9

**Published:** 2024-02-09

**Authors:** Tomonari Hirano, Muneaki Murata, Yurie Watarikawa, Yoichiro Hoshino, Tomoko Abe, Hisato Kunitake

**Affiliations:** 1 Faculty of Agriculture, University of Miyazaki, 1-1 Gakuen-Kibanadai Nishi, Miyazaki, 889-2192 Japan; 2 Nishina Center for Accelerator-Based Science, RIKEN, 2-1 Hirosawa, Wako, Saitama, 351-0198 Japan; 3 Field Science Center for Northern Biosphere, Hokkaido University, Kita 11, Nishi 10, Kita-ku, Sapporo, 060-0811 Japan

**Keywords:** Chromosomal rearrangement, Double fertilization, Embryo, Endosperm, Heavy-ion beam, Pollen

## Abstract

**Key message:**

In *Cyrtanthus mackenii*, development of embryo and endosperm were differentially affected by fertilization of male gametes with DNA damage and mutations.

**Abstract:**

Pollen irradiation with ionizing radiations has been applied in plant breeding and genetic research, and haploid plant induction has mainly been performed by male inactivation with high-dose irradiation. However, the fertilization process of irradiated male gametes and the early development of embryo and endosperm have not received much attention. Heavy-ion beams, a type of radiation, have been widely applied as effective mutagens for plants and show a high mutation rate even at low-dose irradiation. In this study, we analyzed the effects of male gametes of *Cyrtanthus mackenii* irradiated with a carbon-ion beam at low doses on fertilization. In immature seeds derived from the pollination of irradiated pollen grains, two types of embryo sacs were observed: embryo sac with a normally developed embryo and endosperm and embryo sac with an egg cell or an undivided zygote and an endosperm. Abnormalities in chromosome segregation, such as chromosomal bridges, were observed only in the endosperm nuclei, irrespective of the presence or absence of embryogenesis. Therefore, in *Cyrtanthus*, embryogenesis is strongly affected by DNA damage or mutations in male gametes. Moreover, various DNA contents were detected in the embryo and endosperm nuclei, and endoreduplication may have occurred in the endosperm nuclei. As carbon-ion irradiation causes chromosomal rearrangements even at low doses, pollen irradiation can be an interesting tool for studying double fertilization and mutation heritability.

**Supplementary Information:**

The online version contains supplementary material available at 10.1007/s00497-024-00496-9.

## Introduction

Effects of irradiation with ionizing radiation, mainly X-ray and γ-ray, on pollen grains have been studied for a long time, and pollen grains are generally regarded as highly resistant to radiation. Extremely high doses of irradiation are required to inhibit pollen germination and pollen tube elongation (Reviewed in Stephan 2021). In contrast, high-dose irradiation causes chromatin fragmentation in the nuclei of male gametes, and abnormal seed development, such as parthenogenesis and autonomous endosperm formation, has been observed after pollination of irradiated pollen grains (Rojek and Ohad 2023; Sestili and Ficcadenti 1996). These properties have been used in plant breeding, and high-dose irradiated pollen grains have been used to induce haploid lines in many plants. Pollen irradiation has also been applied to the production of seedless fruits (Sugiyama and Morishita 2000). However, details of the fertilization process of irradiated male gametes and early development of the embryo and endosperm have not received much attention and are largely unclear.

A heavy-ion beam, one of the ionizing radiations, comprising ions heavier than helium ions, accelerated nearly to the speed of light by an accelerator has been used as a mutagen for plant breeding. The radiations are categorized based on the value of linear energy transfer (LET; keV/µm). X-ray (2.0–5.0 keV/µm) and γ-ray (0.2 keV/µm) are categorized as low-LET radiation. Heavy-ion beams are categorized as high-LET radiation, and the LET values of the ions used for biological research range from 22.5 to 4000 keV/µm in the RIKEN RI-beam factory (RIBF) (Ryuto et al. 2008). Heavy-ion beams are more biologically effective than X-rays or γ-rays, showing high mutation frequency at low-dose irradiation and induction of a wide range of mutant phenotype (Abe et al. 2021; Tanaka et al. 2010). In *Arabidopsis thaliana*, the size and complexity of mutations induced by heavy-ion beams increase with increasing LET values (Hirano et al. 2012; 2015; Kazama et al. 2011; 2017; Shikazono et al. 2005). Carbon-ion beams at 22.5 and 30 keV/µm primarily induced base substitutions and small size of deletions and insertions (Kazama et al. 2011; 2017), whereas irradiation of argon- and carbon-ion beams at 290 keV/µm caused induction of large deletions and chromosomal rearrangements (Hirano et al. 2012; 2015).

Heavy-ion beam irradiation has been applied to various plant materials including pollen for mutant induction and genetic analyses. Hermaphroditic and asexual mutants have been successfully screened in a dioecious plant, *Silene latifolia*, by pollinating pollen grains irradiated with heavy-ion beams (Kazama et al. 2016). Naito et al. (2005) reported mutation heritability using pollen grains irradiated with heavy-ion beams, and the small deletions were normally transmitted to the next generation, whereas the large deletions were not. In *Cyrtanthus mackenii*, DNA damage responses in male gametes of heavy-ion beam-irradiated pollen were investigated. *C. mackenii* forms bicellular pollen, and the generative cell divides into two sperm cells during pollen tube growth. Irradiated generative cells with DNA double-strand breaks (DSBs) are arrested at metaphase, and the DSBs are repaired (Hirano et al. 2013; 2021). Sperm cells passing through metaphase show abnormalities in chromosomal separation, and chromosomal bridges are formed frequently. In particular, generative cell-like sperm cells (GC-like SCs), defined as cells with generative cell-like nuclei and sperm cell-like microtubule arrays that completed pollen mitosis (PM) II but failed to separate chromosomes, were observed (Hirano et al. 2013). These results indicated that male gametes have a mechanism to repair DNA damage and transmit genomic information to the next generation.

To understand the mechanisms that maintain genome stability and mutation selection during the double fertilization process, it is necessary to investigate the fertilization of gametes with DNA damage or mutations and the processes of embryo and endosperm development. Therefore, in this study, male gametes irradiated with carbon-ion beam at low doses were fertilized, and then, the embryo and endosperm development were analyzed.

## Materials and methods

### Plant materials

Anthers of *Cyrtanthus mackenii* Hook. f. (Amaryllidaceae) were collected in 0.2-mL tubes and maintained at –20 °C after dehiscence. The anthers were irradiated with carbon-ion beam (22.5 keV/μm) at absorbed doses of 10 and 40 Gy using the E5 beam line in RIBF and then stored at –20 °C until further analysis. Pollen grains irradiated at 0 Gy and 40 Gy were stained with 1 μg/mL of Hoechst 33,258 and 0.1% (v/v) polyoxyethylene (20) sorbitan monolaurate in pollen germination medium (Hirano and Hoshino 2010) for 2 h and were then observed under an optical microscope (BX51-34-FL-2; OLYMPUS). The flower buds were emasculated and bagged for crossing. Two days after emasculation, unirradiated and irradiated pollen grains were pollinated. The pistils were collected at 2 and 14 days after pollination (DAP) and fixed in a fixative of formaldehyde, acetic acid, and ethanol (FAA) or 4% paraformaldehyde (PFA) in phosphate-buffered saline for 24 h. The fixed pistils were replaced with 70% ethanol and stored at 4 °C.

### Pollen tube growth in pistil

The pistils fixed in FAA at 2 DAP were hydrolyzed in 1 M NaOH at 60 °C for 20 min. After washing thrice with distilled water, the pistils were stained with a 0.2% aniline blue solution overnight. Pollen tubes in the pistil were then observed under the optical microscope.

### Cytological observation of the embryo sac

Paraffin sectioning was performed on embryo sacs fixed in FAA at 14 DAP, according to Park et al. (2023). The samples were sectioned to a thickness of 10 µm using a microtome and stained with hematoxylin. Specimens were observed and photographed using the optical microscope. After observation, the immature seeds were categorized based on their embryo and endosperm development.

For embryo sac isolation from the seeds, ovaries fixed in PFA at 14 DAP were replaced with PBS for 1 day. Enlarged seeds were removed from the ovaries under a stereomicroscope (SZ2-ILST; OLYMPUS) and dissected using tweezers and glass needles. The isolated embryo sacs were stained with 1 μg/mL of Hoechst 33,258 and 0.1% (v/v) polyoxyethylene (20) sorbitan monolaurate in PBS for 30 min and mounted on slide grass in an antifade reagent (SlowFade Gold Antifade Mountant, S36937, Thermo Fisher Scientific). Images were obtained at 1.0 μm steps along the Z-axis using a confocal laser scanning microscope (LSM 700, Carl Zeiss). All the Z-stack images were observed to confirm the embryo development.

### Relative fluorescence between embryo and endosperm nuclei

To estimate the fluorescence intensity arising from Hoechst 33,258 in the embryo and endosperm nuclei, the total fluorescence emitted from each nucleus in the embryo sacs was measured by integrating the volume and mean fluorescence over the entire image in Z-stacks. ImageJ software (Schneider 2012) was used for the calculations. The net nuclear fluorescence intensity was calculated by subtracting the total background fluorescence around each embryo sac from the total measured fluorescence of each nucleus.

### Flow cytometry analysis

Imbibed seeds without seed coats were chopped with a sharp razor blade in 200 µL of the Otto I buffer (Otto 1990) and were filtered through a layered 30 µm nylon mesh. The filtrates were then mixed with 800 mL of DAPI staining buffer (Mishiba et al. 2000) and analyzed using a CyFlow Ploidy Analyser (Sysmex Corporation, Kobe, Japan).

## Results

### Fertilization of the carbon-ion irradiated male gametes

**Fig. 1:**
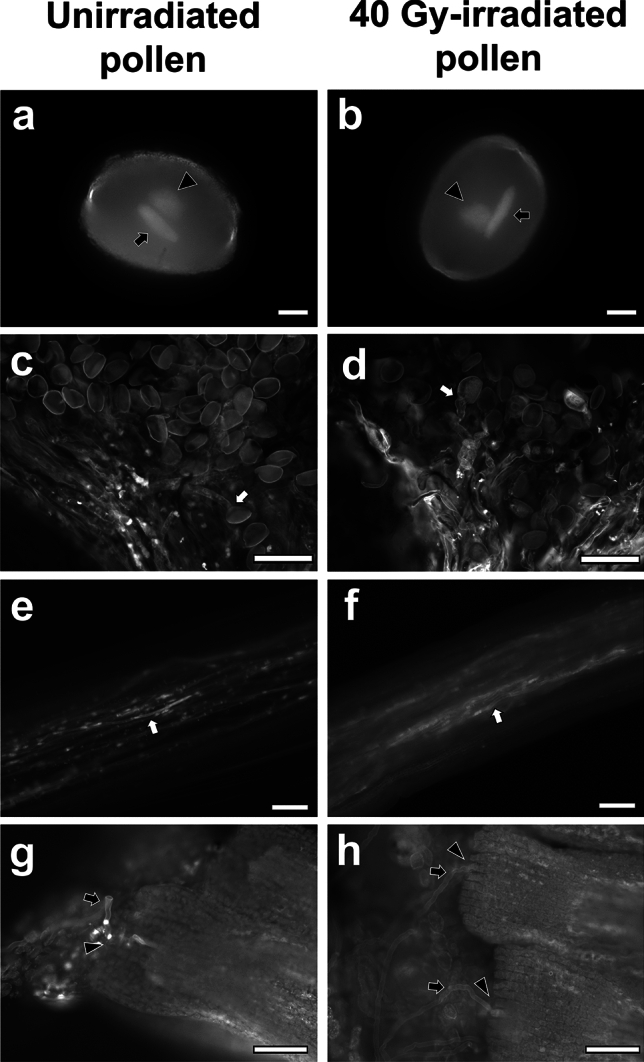
Pollen tube growth in pistils. Pollen grains irradiated at 0 Gy (**a**) and 40 Gy (**b**) were stained with Hoechst 33,258. The arrows and arrowheads indicate generative cell nuclei and vegetative nuclei, respectively. The pollen tubes derived from the unirradiated (**c**, **e**, **g**) and irradiated pollen grains (**d**, **f**, **h**) were stained with aniline blue. The pollen grains germinated on the stigma (**c**, **d**). The arrows indicate the germinated pollen grains. The pollen tubes elongated in the style (**e**, **f**) and penetrated the ovules (**g**, **h**). The arrows and arrowheads indicate the pollen tubes and micropyle regions in the ovules, respectively. Bars = 10 µm (**a**, **b**), 100 µm (**c**–**h**)

First, we confirmed pre-germination state of pollen grains using DNA staining. *C. mackenii* forms bicellular pollen, and vegetative nucleus and generative cell nucleus were observed in each unirradiated pollen grain (Fig. 1a). The irradiated pollen grains also harbored the vegetative nuclei and generative cell nuclei (Fig. 1b). Pollen tube growth after pollination at 2 DAP was observed using aniline blue staining. Both pollen tubes derived from pollen grains, with or without irradiation, were elongated in style and reached the ovules (Fig. 1c–h).

**Fig. 2:**
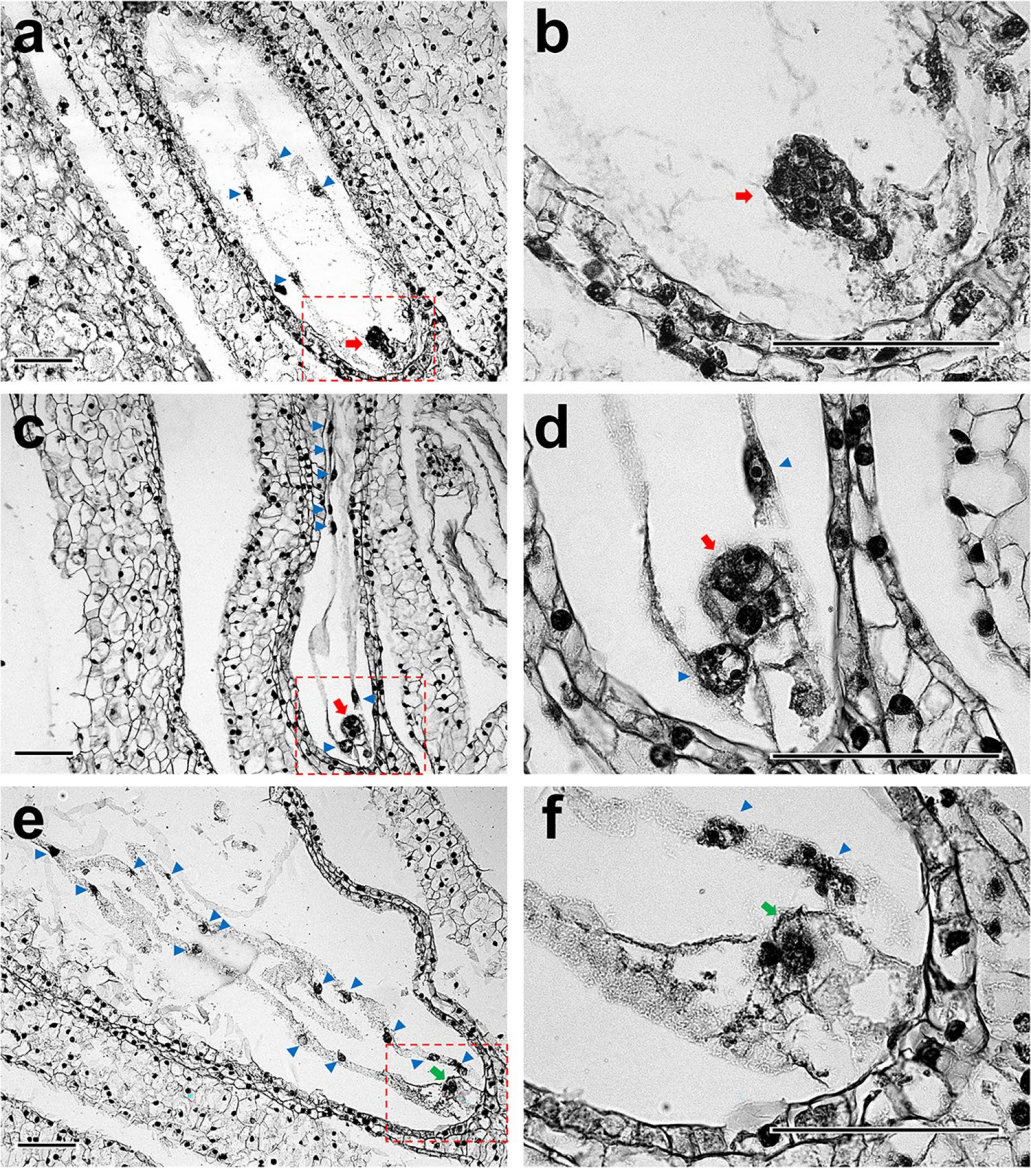
Longitudinal section of embryo sacs at 14 DAP. Embryo sac images (**a**, **c**, **e**) and their respective magnified images for the embryos (**b**, **d**, **f**), which coincides with the area enclosed by red dotted line in the embryo sac images, are shown. The embryo sac with an embryo and an endosperm after the pollination of the unirradiated pollen grains (**a**). The embryo sac with an embryo and an endosperm after the pollination of 40 Gy-irradiated pollen grains (**c**), and the embryo sac with an egg cell/zygote and an endosperm after the pollination of 40 Gy-irradiated pollen grains (**e**). The red and green arrows indicate the embryo cells and an egg cell/zygote cell, respectively. The blue arrowheads indicate the endosperm nuclei. Bars = 100 µm

To investigate the effects of fertilization with carbon-ion beam-irradiated male gametes on embryo and endosperm formation, the embryo sacs in immature seeds at 14 DAP were histologically observed. When non-irradiated pollen grains were pollinated, embryo sacs containing an embryo and an endosperm with a syncytium structure were observed and categorized as normal (Fig. 2a, b). In contrast, after pollination of the 40 Gy-irradiated pollen grains, embryo sacs with an egg cell or an undivided zygote and a syncytium endosperm were observed in addition to normal embryo sacs (Fig. 2c–f). Moreover, some of the embryo sacs derived from pollination of the irradiated pollen grains showed a narrowed structure (Fig. 2c, d). The observed embryo sacs were categorized based on embryo and endosperm formation (Table 1). In fertilization of irradiated male gametes, 2% (1/58) and 14% (11/77) of the embryo sacs showed no embryo development at 10 and 40 Gy-irradiated pollen grains, respectively, and the number of embryo sacs without embryo development increased in a dose-dependent manner. No embryo sacs without endosperm development were observed.

**Table 1:** Effect of pollination of C-ion irradiated pollen grains on embryo and endosperm development in the embryo sacs of the immature seeds at 14 DAP

Dose (Gy)	Types of embryo sac			
	Normal	Without embryo	Without endosperm	Total
0	67	0	0	67
10	57	1	0	58
40	66	11	0	77

### Cytological observation of embryo sacs isolated from immature seeds

**Fig. 3:**
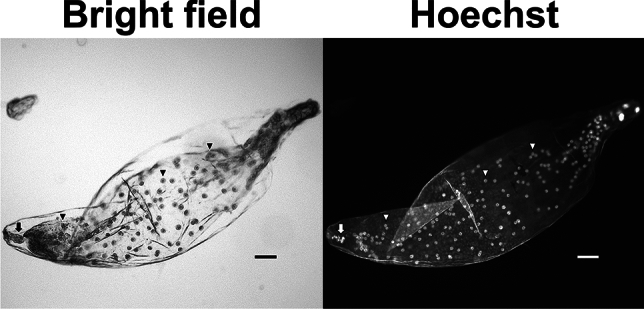
Whole images of embryo sac isolated from immature seed fixed with PFA at 14 DAP. The nuclei were stained with Hoechst 33,258. The arrows and arrowheads indicate the embryo cells and endosperm nuclei, respectively. Bars = 100 µm

**Fig. 4:**
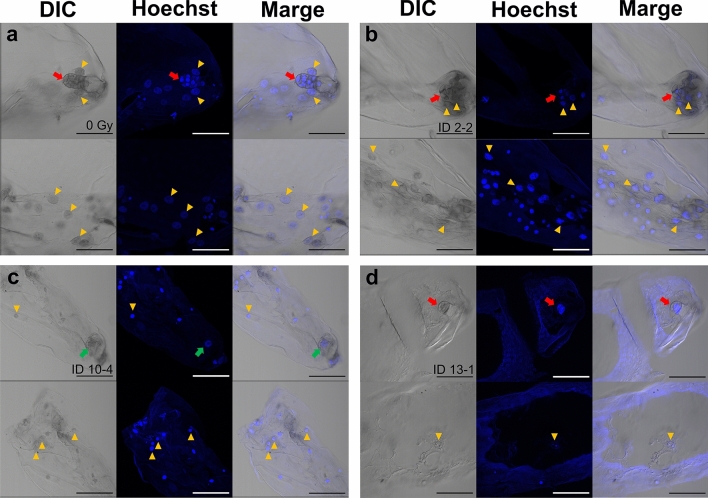
Embryo or egg cell/zygote and endosperm in isolated embryo sac at 14 DAP. The embryo sacs isolated from PFA-fixed immature seeds, and nuclei stained with Hoechst 33,258. Embryo and endosperm derived from pollination of 0 Gy-irradiated pollen grains (**a**). Embryo and endosperm (embryo sac ID 2–2) derived from pollination of 40 Gy-irradiated pollen grains (**b**). Egg cell/zygote and endosperm (ID 10–4) derived from pollination of 40 Gy-irradiated pollen grains (**c**). The embryo sac derived from pollination of 40 Gy-irradiated pollen grains (ID 13–1) shows that the embryo nuclei are larger than the endosperm nuclei (**d**). The red and green arrows indicate the embryo cells and an egg cell/zygote cell, respectively. The yellow arrowheads indicate the endosperm nuclei. Bars = 100 µm

**Fig. 5:**
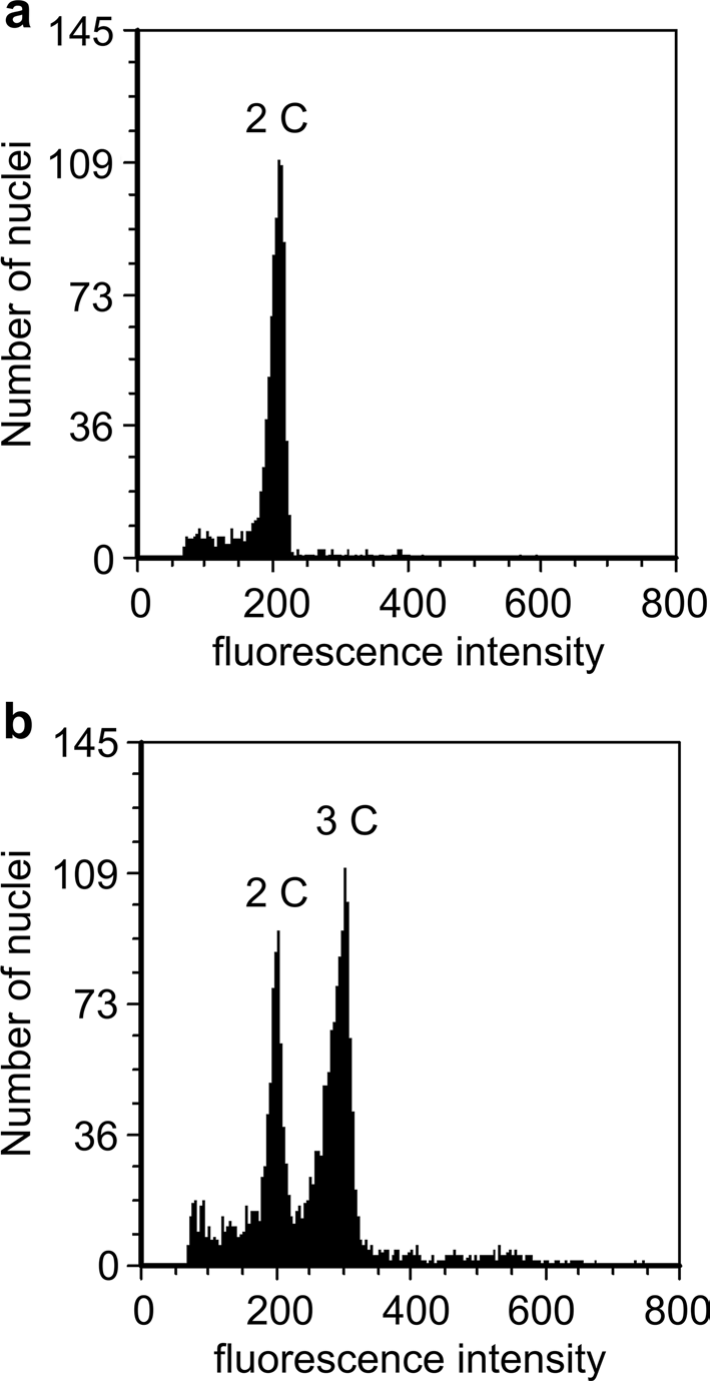
Histograms of flow cytometry analysis in leaf and mature seed. Fluorescence intensity of nuclei from the leaf as a standard for diploid tissue (**a**) and from mature seed containing embryo and endosperm (**b**)

To elucidate the details of the fertilization process of irradiated male gametes and subsequent seed development, DNA content of embryo and endosperm nuclei was compared. At first, embryo sacs were isolated from the PFA-fixed seeds at 14 DAP under a microscope for detailed analysis of the embryo and endosperm (Fig. 3). Nuclei in the isolated embryo sacs were stained with Hoechst 33,258 and the fluorescence intensities of the embryo and endosperm nuclei were measured for estimation of DNA content. In the pollination of the unirradiated pollen grains, the isolated embryo sacs contained 3–13-cell embryos and an endosperm with a syncytium structure (Fig. 4a, Table S1), and the ratio of the relative fluorescence intensity in the embryo and endosperm nuclei was approximately 1:1.8 (Table 2). The fluorescence intensity in seed nuclei was analyzed using flow cytometry and showed 2C peak which was almost the same as that in the leaf nuclei (2C) and 3C peak (Fig. 5). Although the embryo and endosperm were not separated in the analysis, the embryo and endosperm nuclei were basically considered to show the 2C and 3C peaks, respectively. Therefore, the measurement of the fluorescence intensity of the embryo and endosperm nuclei in the isolated embryo sac is effective for calculating the DNA content ratio.

**Table 2:** Ratio of relative fluorescence intensity between embryo (egg cell/zygote) and endosperm nuclei in isolated embryo sacs at 14 DAP derived from pollination of 40 Gy-irradiated pollen grains

Embryo sac ID	Embryo formation	Relative fluorescence intensity (mean ± SE)				
		Em (Eg)	End	End Max	Em (Eg): End	Nuclear division in the endosperm
0 Gy*	+	100 ± 6.9	184.7 ± 7.5	637.2	1: 1.8	Normal
7–2	+	100 ± 11.4	141.7 ± 18.1	227.8	1: 1.4	Normal
13–1		100 ± 7.0	29.1 ± 3.6	44.8	1: 0.3	Normal
15–1		100 ± 3.2	133.0 ± 12.8	327.8	1: 1.3	Normal
18–2		100 ± 16.8	237.8 ± 11.4	444.9	1: 2.4	Normal
2–1		100 ± 37.8	353.1 ± 53.6	1004.8	1: 3.5	Endoreduplication
2–2		100 ± 11.6	279.6 ± 23.0	1017.3	1: 2.8	Endoreduplication
9–2		100 ± 9.3	352.2 ± 50.7	1856.1	1: 3.5	Endoreduplication
9–3		100 ± 16.7	424.1 ± 48.6	1064.3	1: 4.2	Endoreduplication
12–2		100 ± 16.4	443.9 ± 36.2	1376.1	1: 4.4	Endoreduplication
12–3		100 ± 9.9	2761.1 ± 2384.0﻿	12,292.1	1: 27.6	Endoreduplication
6–1	-	100	241.0 ± ﻿18.3	555.3	1: 2.4	Normal
8–1		100	196.8 ± 41.7	538.8	1: 2.0	Normal
8–2		100	284.5 ± 30.8	315.3	1: 2.8	Normal
9–1		100	166.6 ± 11.8	267.7	1: 1.7	Normal
10–4		100	54.7 ± 3.8	91.5	1: 0.6	Normal
13–3		100	67.9 ± 13.1	98.4	1: 0.7	Normal
17–1		100	228.0 ± 41.9	739.3	1: 2.3	Normal
18–3		100	90.6 ± 12.5	316.0	1: 0.9	Normal
18–4		100	144.1 ± 14.6	470.7	1: 1.4	Normal
19–3		100	145.2 ± 21.7	350.9	1: 1.45	Normal
4–2		100	834.2 ± 448.6	1282.8	1: 8.3	Endoreduplication
7–3		100	2703.6 ± 423.1	3893.5	1: 27.0	Endoreduplication
8–4		100	647.9 ± 56.2	1540.3	1: 6.5	Endoreduplication
14–1		100	339.9 ± 29.9	1271.8	1: 3.4	Endoreduplication
16–1		100	437.4 ± 76.0	1105.8	1: 4.4	Endoreduplication

The fluorescence intensity values in the endosperm (End) are relative to the mean values of embryo (Em) fluorescence or the values of the egg cell/zygote (Eg) fluorescence, which are set to 100. *Mean of 5 embryo sacs. End Max; maximum relative fluorescence intensity in the endosperm nuclei

### Effects of fertilization of the carbon-ion irradiated male gametes on the embryo and endosperm development

**Fig. 6:**
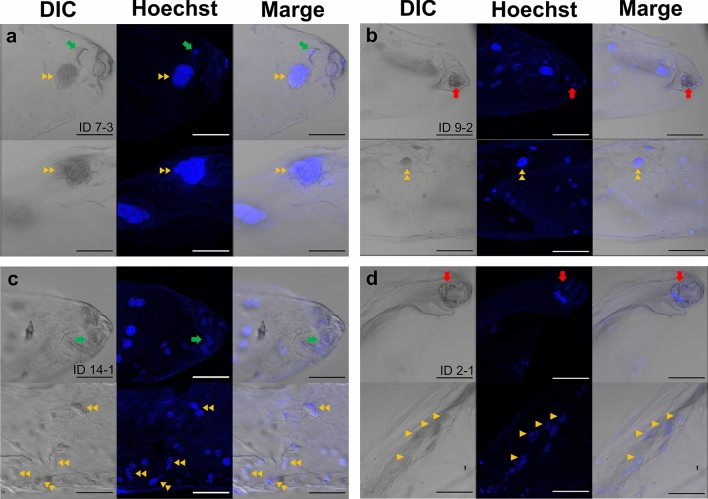
Abnormal endosperm nuclei in isolated embryo sacs derived from pollination of 40 Gy-irradiated pollen grains. Endosperm with only enlarged nuclei (**a**, ID 7–3) and with normal and enlarged nuclei (**b**, ID 9–2). Endosperm nuclei with lagging chromosome and chromosomal bridges (**c**, ID 14–1). Endosperm nuclei at metaphase (**d**, ID 2–1). The red and green arrows indicate the embryo cells and an egg cell/zygote cell, respectively. The double yellow arrowheads indicate the endosperm nuclei with abnormalities, such as the enlarged nuclei, lagging chromosome, and chromosomal bridge. The yellow arrowheads indicate the nuclei at metaphase. Bars = 100 µm

Embryo sac isolation and fluorescence intensity measurements were performed in PFA-fixed seeds derived from irradiated pollen pollination. When zygotes divided at least once, this was considered as the onset of embryo formation, and 10 of the 25 isolated embryo sacs formed embryos, which comprised 2–6 cells (Fig. 4b, Table 2, Table S1). The remaining egg cells and zygotes did not divide (Fig. 4c). As in the pollination of the unirradiated pollen, fluorescence intensity ratios close to 1:1.8 were found in both embryo-formed and non-embryo-formed embryo sacs, but many embryo sacs showed characteristic ratios (Table 2). The relative fluorescence intensity of the embryo nuclei was greater than that of the endosperm nuclei in embryo sac 13–1 with the embryo (Fig. 4d) and those in 10–4 (Fig. 4c), 13–3, and 18–3, which did not form embryos. Moreover, enlarged nuclei were observed in the endosperm, and the following two types of embryo sacs were observed: all endosperm nuclei were enlarged (Fig. 6a) and only some endosperm nuclei were enlarged (Fig. 6b). In addition to the variation in the fluorescence intensity ratio, abnormalities in chromosomal separation in the endosperm nuclei were observed (Fig. 6c, d). Lagging chromosomes and chromosomal bridges were detected and incomplete division of the nuclei was observed. There was an embryo sac with numerous endosperm nuclei at metaphase (Fig. 6d).

## Discussion

Studies using pollen grains irradiated with low-LET radiation were mainly aimed at inactivating male gametes and thus applied extremely high doses. In contrast, in the present study, *Cyrtanthus* pollen grains were irradiated with carbon-ion beams at low doses, which is several tenths of that used in previous studies. This study investigated the effects of male gamete fertilization on embryo and endosperm development and revealed several characteristic changes.

In the effects of carbon-ion beam irradiation on *Cyrtanthus* pollen grains, the irradiation up to 80 Gy had no effect on germination and in vitro pollen tube elongation (Hirano et al. 2013). In this study, we confirmed that pollen tubes from 40 Gy-irradiated pollen grains were elongated in style and reached the ovules (Fig. 1), suggesting that carbon-ion beam irradiation does not significantly inhibit pollen tube elongation, even under *in vivo* conditions.

In this study, immature seeds with unfertilized egg cells or zygotes with arrested cell division were observed after pollination with irradiated pollen grains (Figs. 2, 4, and 6). Autonomous endosperm formation has been reported in several plant species (Rojek and Ohad 2023; Sestili and Ficcadenti 1996). In kiwifruit (*Actinidia deliciosa*), pollination with γ-ray irradiated pollen induced seeds with endosperm only or endosperm and embryo, and the only developed endosperm were autonomously developed (Musial and Przywara 1998; 1999). Therefore, autonomous endosperm formation is possibly one of the causes of seed development in *Cyrtanthus*, similar to that in kiwifruit. If the irradiated male gametes were unfertilized, the ratio of the fluorescence intensity of the egg nucleus to that of the endosperm nuclei would be 1:2, and embryo sacs 6–1, 8–1, 8–2, 17–1 would fit the pattern (Table 2). Pollen tube-dependent ovule enlargement morphology (POEM), in which pollen tubes reach the embryo sac and release their contents, stimulates endosperm development without fertilization, has been reported in *A. thaliana* (Kasahara et al. 2016). Therefore, the involvement of POEM in *Cyrtanthus* is worth investigating.

Three patterns of the seeds with embryo and endosperm, embryo only, and endosperm only were observed after pollination with γ-ray irradiated pollen in apple, and the embryo and endosperm development inhibited with increasing absorbed dose (Nicoll et al. 1987; Zhang and Lespinasse 1991). In the immature seeds with the embryo in this study, mean number of the embryo nuclei in 0 Gy immature seeds was 7.2 ± 1.7 (*n* = 5, mean ± SE, Table S1). However, that in 40 Gy immature seeds was 2.9 ± 0.5 (n = 10, mean ± SE, Table S1). Therefore, it is suggested that cell division in the embryo is arrested owing to the transmission of DNA damage or mutations derived from the male gametes. Even low-dose irradiation with heavy-ion beams activates the spindle assembly checkpoint during PMII (Hirano et al. 2013; 2021). Checkpoints are probably activated by their transmission to the zygote through fertilization, resulting in the arrest of the zygote cell cycle.

Male gametes irradiated with carbon-ion beams have been reported to undergo unequal division at PMII and form unreduced sperm cell, GC-like SC (Hirano et al. 2013). In some immature seeds, the fluorescence intensity of the embryo nuclei or egg/zygote nuclei exceeded that of the endosperm nuclei (Table 2), suggesting that a GC-like SC or a sperm cell with high DNA content in the unequal division were fertilized with an egg cell. In *cdc2a*/*cdka-1* mutants of Arabidopsis, a single generative (sperm)-like cell is formed instead of the two sperm cells in the mutant pollen (Imakawa et al. 2006; Nowack et al. 2006). The mutant cell fertilizes the egg cell and autonomous endosperm development is observed. If GC-like SCs in *Cyrtanthus* can be fertilized with egg cells, autonomous endosperm development may be induced. Moreover, in 10–4, 13–3, and 18–3, cell division of the zygotes was possibly arrested. In contrast, another pattern, in which a GC-like SC fuses with a central cell, is possible. In this pattern, the relative fluorescence intensity of the endosperm nuclei was assumed to be 4–8 times higher than that of the egg nuclei, which may be suitable for some embryo sacs.

The enlarged endosperm nuclei showed very high fluorescence intensities (Fig. 6; Table 2), suggesting that endoreduplication had occurred. In this study, endoreduplication was defined as the maximum fluorescence intensity of the endosperm nuclei, which was more than eight times the relative fluorescence intensity of the embryo and egg/zygote nuclei. Since the maximum fluorescence intensity of the endosperm nucleus due to the fertilization of irradiated male gametes is assumed to occur when the GC-like SC (2C) fertilizes the central cell (2C) to form the endosperm, the relative fluorescence intensity of the G2 phase of the endosperm nucleus is eight times higher than that of the G1 phase of the egg nucleus. Therefore, any fluorescence intensity exceeding this value was considered to be due to endoreduplication. According to this definition, endoreduplication of the endosperm nuclei was confirmed in 11 embryo sacs, regardless of the occurrence of embryogenesis (Table 2). Grasses such as rice and maize are characterized by the transition of endosperm nuclei to the endoreduplication cycle after the completion of cell division (Sabelli 2012). However, it is clear from the results of the flow cytometric analysis of mature seeds that endoreduplication does not occur during normal endosperm development in *Cyrtanthus* (Fig. 5). Metaphase nuclei were frequently observed in the endosperm of embryo sac 2–1, and the presence of chromosomal bridges was also confirmed (Fig. 6d). In addition, the endosperm nuclei of 14–1 could not divide or fuse (Fig. 6c). These abnormalities are similar to those observed in the PMII of male gametes (Hirano et al. 2013; 2021). Therefore, it is considered that the male gamete nuclei fuse with the polar nuclei, and that DNA damage and mutations in the male gametes are inherited by the endosperm nuclei and are caused by endoreduplication by repeating the cell cycle with incomplete nuclear division. In contrast, no chromosomal aberrations were observed in the embryo nuclei. This suggests that cell cycle checkpoints in the zygote are stricter than those in the endosperm and that mitosis does not proceed in the presence of DNA damage or abnormalities in chromosome segregation.

Whole-genome analyses have revealed that irradiation with C- and Ar-ion beams induces deletions and chromosomal rearrangements in the genome (Hirano et al. 2015; Ichida et al. 2019; Kazama et al. 2017; Morita et al. 2021). However, the transmissibility of these mutations is not fully understood. It is important to know the details of the fertilization process to determine whether a mutation has been passed on to the next generation, and a series of studies, including irradiation of pollen, pollen tube elongation, fertilization, and embryogenesis, are required (Hirano et al. 2022). The low-dose irradiation used in this study caused chromosomal rearrangements but was expected to induce relatively few genome-wide mutations. Therefore, a comprehensive set of analyses established in *Cyrtanthus* would be useful for studying genome stability during double fertilization and mutation heritability.

## Supplementary Information

Below is the link to the electronic supplementary material.

Supplementary file1 (XLSX 11 KB)
